# Innovations in online classes introduced during the COVID-19 pandemic and their educational outcomes in Japan

**DOI:** 10.1186/s12909-023-04874-1

**Published:** 2023-11-22

**Authors:** Kyoko Yamamoto, Kumiko Akiyoshi, Hidekazu Kondo, Hidefumi Akioka, Yasushi Teshima, Kunio Yufu, Naohiko Takahashi, Mikiko Nakagawa

**Affiliations:** 1 Medical Education Center, Faculty of Medicine, Oita University, 1-1, Idaigaoka, Hsama-Machi, Yufu, Oita 879-5593 Japan; 2 Department of Cardiology and Clinical Examination and Diagnosis, Faculty of Medicine, Oita University, 1-1, Idaigaoka, Hsama-Machi, Yufu, Oita 879-5593 Japan

**Keywords:** Medical, Education, Students, COVID-19, Online, Class, Pandemic

## Abstract

**Background:**

The novel coronavirus disease (COVID-19) pandemic emerged in Japan in February 2020, forcing the adoption of online education by university medical schools across Japan. The advantages and disadvantages of online education have been studied in Japan; however, the educational outcome of online classes conducted during the COVID-19 pandemic has not been completely evaluated. In this study, we examined the relationship between lecture format (e.g., face-to-face or online) and performance of third-year university students in their organ-specific cardiovascular course examination.

**Methods:**

This retrospective, nonclinical, and noninterventional comparative educational study included 550 third-year medical students who took a cardiovascular course between April 2018 and May 2022. Cardiovascular coursework was conducted in-person in 2018 and 2019, online in 2020 and 2021, and again in-person in 2022. The course comprised 62 lecture and 2 problem-based learning (PBL) sessions. A quiz was set up in advance on Moodle based on all lectures conducted in 2021 and 2022. A written examination was administered at the end of the course to evaluate the knowledge of students. The student online course evaluation questionnaires were administered in 2020 and 2021. Examination scores and proportion of failures in each year were compared.

**Results:**

The mean examination scores were significantly higher in 2021 and 2022 than in 2018, 2019, and 2020 (*p* < 0.05). Univariate and multivariate analyses adjusted for the class type, online quiz, and PBL revealed that only online quiz was significantly associated with better examination results (*p* < 0.05). A student course evaluation survey indicated that the online format did not interfere with the students’ learning and was beneficial.

**Conclusions:**

The introduction of online classes into medical education due to the COVID-19 pandemic was as effective as face-to-face classes owing to learning management system and other innovations, such as online quizzes. Online education may confer more benefits when provided in a combination with face-to-face learning after COVID-19 pandemic.

## Background

The novel coronavirus disease (COVID-19) pandemic forced medical schools worldwide to shift their learning methods toward online learning; the experience of this transformation has been analyzed by several researchers [1–14]. Although online programs were generally well accepted by medical students, reports have shown varying student satisfaction levels with online education [2, 9, 10, 12].

In Japan, the COVID-19 pandemic emerged in February 2020, forcing university medical schools across Japan to completely adopt online education. Studies on medical education during pandemics have revealed that online education is useful for saving time and creating a flexible learning environment, but it also has several important drawbacks, including internet problems, nonstandardized teaching methods, potential student insecurity, and lack of clinical practice experience [15–17]. However, the impact of online education on student outcome achievement has not been completely evaluated.

The medical education center of Oita University quickly coordinated with education coordinators to ensure smooth transition to an online learning environment for undergraduate courses. Moodle is a learning management system (LMS) that has been used since 2017. Because of the COVID-19 pandemic, online classes via Moodle were conducted for the entire 2020 curriculum using two methods: real-time interactive classes via Zoom or on-demand classes with audio recordings of the class content. All online class materials were uploaded to Moodle. Face-to-face lectures were conducted for some practical classes in 2021 and almost all classes in 2022. The curriculum for third-year medical students comprises 15 organ-specific courses, including the cardiovascular course. A written examination is administered at the end of each course to assess knowledge level of the students.

In this study, we examined the relationship between the class format (e.g., face-to-face or online) and performance of students in the cardiovascular course examination to determine the educational outcome of online classes conducted during the COVID-19 pandemic.

## Methods

### Study design

This was a retrospective, nonclinical, and noninterventional comparative educational study. A total of 550 third-year medical students who took the cardiovascular course between April 2018 and May 2022 were enrolled in the study. The cardiovascular course was conducted by 27 faculty members from the physiology, biochemistry, cardiovascular internal medicine, cardiovascular surgery, pediatric cardiovascular disease, radiology, endocrinology and metabolism, and public health departments. This course comprises 62 lecture sessions and 2 small-group study and presentation sessions in PBL. Table 1 summarizes the changes in the class format of the cardiovascular course. Cardiovascular coursework was conducted in-person in 2018 and 2019 before the COVID-19 pandemic, online in 2020 and 2021, and in-person in 2022.

**Table 1:** Class format of the cardiovascular course

Year	Class type	Online quiz	PBL
2018	In-person	No	Yes (in-person)
2019	In-person	No	Yes (in-person)
2020	Online	No	No
2021	Online	Yes	Yes (online)
2022	In-person	Yes	Yes (online)

*PBL* problem-based learning

### Ethics approval and consent to participate

The ethics committee of Oita University approved our study and informed consent was obtained from all participants through an opt-out methodology.

### PBL

A small-group discussion on PBL was conducted online in 2021 and 2022 via Zoom using breakout rooms. The PBL presentation sessions were conducted online in 2021 and face-to-face in 2022. Unfortunately, the PBL sessions were cancelled in 2020 because of the lack of time to prepare online PBL sessions.

### Quiz

For all lectures conducted in 2021 and 2022, a quiz was set up in advance in Moodle. The quiz comprised multiple-choice questions enquiring the most important content of each lecture. Students answered the quiz after each lecture, and their scores and feedback were provided on Moodle. In 2022, students attempted a quiz on Moodle after attending a face-to-face lecture, and the faculty members provided an on-site explanation of the quiz.

### Examination

Each year after the completion of the cardiovascular course, a written examination was conducted in which questions were asked in various formats, such as multiple-choice, true–false, and essay questions. The difficulty level of each year’s questions was almost consistent. Examination scores were presented on a 100-point scale, and the mean score for the academic year was calculated. Students who scored less than 60 were considered failed students.

### Course evaluation survey

After each course, the students completed an online course evaluation survey each year through the university’s web-based educational information system. In 2020 and 2021, two brief questions about online classes were added to the survey: Question 1: Has the online format interfered with your learning? and Question 2: Did you see the benefits of the online format of the class? The students were requested to freely state their opinions in each question. In this study, we analyzed the results of class evaluation questionnaires for all third-year courses in 2020 and 2021 to assess students’ opinions on online classes.

### Statistical analysis

JMP Statistics (version 13.2.0; SAS Institute, Cary, NC, USA) was used for data analysis. Considering that cardiovascular course is mandatory for third-year students, all students answered the quizzes, participated in PBL sessions, and took the examinations. Therefore, there were no missing data in this analysis.

We compared the examination scores and proportion of failures in each year. Data are presented as mean ± standard deviation. We used analysis of variance and the Bonferroni post-hoc test to evaluate differences between examination results obtained in the studied years. Univariate and multivariate logistic regression analyses were performed to identify independent predictors of the examination outcome. A *p* value of < 0.05 was considered statistically significant.

## Results

Table 2 shows the results of the 2018–2022 examinations. The mean exam scores were significantly higher and significantly fewer students failed the exam in 2021 and 2022 than in 2018, 2019, and 2020 (*p* < 0.05).

**Table 2:** Results of examination

Year	n	Test scores	Failure (%)
2018	113	70.1 ± 10.9	21 (18.6)
2019	113	72.7 ± 11.7	10 (8.8)
2020	106	71.9 ± 12.6	15 (14.2)
2021	106	78.5 ± 9.8*	4 (3.8) *
2022	112	75.8 ± 9.9*	5 (4.5) *

Values are mean ± SD or n (%)

^*^p < 0.05 vs 2018, 2019, and 2020

Factors that influenced students’ examination performance included the mode of instruction (face-to-face or online), administration of quizzes, and use of PBL. Univariate and multivariate analyses adjusted for the class type, online quiz, and PBL revealed that only the administration of online quiz was significantly associated with the favorable results of examination (Table 3).

**Table 3:** Univariate and multivariate logistic regression analyses of the results of examination

Variables	Univariate *P* value	Multivariate		
		**OR**	**95% CI**	***P* value**
**Class type (online)**	0.7975	1.192	0.311–4.563	0.7980
**Online quiz (yes)**	0.0055*	3.402	1.285–9.007	0.0137*
**PBL (yes)**	0.7817	1.236	0.276–5.527	0.7820

*PBL* problem-based learning, *OR* odds ratio, *CI* confidence interval

^*^p < 0.05

**Fig. 1:**
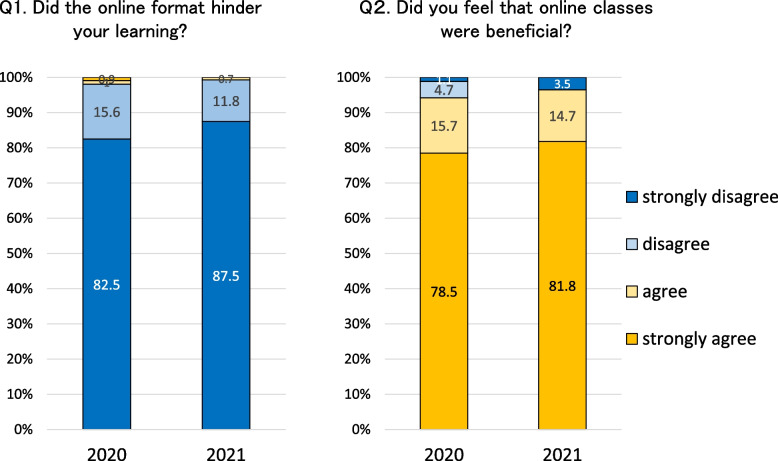
Student Evaluations of Online Classes. Data from class evaluation questionnaires answered by third-year students in 2020 and 2021 (total number of respondents: 641 and 147, respectively). Numbers in the graphs indicate percentages

Figure 1 shows the answers for the questions about online classes that were added in the student online course evaluation survey conducted in 2020 and 2021.

For Question 1 (“Did the online format hinder your learning?”), almost all students answered that it did not interfere with their learning; however, a few students stated that they occasionally faced difficulty connecting to Zoom. Approximately 95% students positively responded to Question 2 (“Did you feel that online classes were beneficial?”). Many students cited “being able to concentrate in the comfort of their own home” and “on-demand classes allow them to take the class at their own pace and review it as many times as they want” as the advantages of online classes.

## Discussion

As the new academic year for school education in Japan starts in April, each university made a rapid transition to online classes to prepare for the new semester in 2020. As Oita University had already been using LMS, Moodle, the setup of online classes proceeded relatively smoothly. In addition, under the supervision of the university’s Education Management Office, Zoom was quickly introduced into the education system, and training sessions were held for faculty and staff. Technical and financial support provided to students was also strengthened. However, a few students had difficulty in accessing the internet, thereby necessitating further improvement in the internet connectivity in the campus and at home.

The content and curriculum quantity of online classes were same as those of in-person classes, and it was ensured that the quality of education was not compromised. In the cardiovascular course, PBL was conducted online in 2021 and 2022, with small-group discussions and presentations, which were conducted without any particular inconvenience compared with face-to-face classes. Quizzes were administered on Moodle in all classes for the first time in 2021, which also served as a way to record class attendance. This approach was continued in 2022 even when the classes were conducted in-person.

In the cardiovascular course, video teaching materials are extensively used in the lectures, particularly for teaching echocardiography, coronary angiography, cardiac surgery, catheter-based treatment procedures, and cardiac rehabilitation. Active learning teaching methods such as video materials, PBL, and quizzes can be implemented in online classes and in face-to-face classes. Furthermore, the use of LMS allows students to view lecture materials again and review quizzes, thereby increasing the learning efficiency of online classes. Compared with classes conducted in large classrooms, online classes provided opportunities for students to concentrate on the lectures in a quiet environment at home. As evident in the results of the class evaluation questionnaire, the students were highly satisfied with online classes (Fig. 1).

The performance in the written examination conducted in 2020 and 2021, i.e., when classes were conducted online, was the same as that in examinations conducted in 2018 and 2019, i.e., when classes were conducted in-person. The performance in the written exam significantly increased in 2021 (Table 2). This increase was attributed to the administration of quizzes regardless of the class format (face-to-face or online) and PBL (Table 3). The setting up and evaluation of quizzes on the LMS was not time consuming for the instructors, and we intend to continue this practice in the future.

The COVID-19 pandemic forced medical schools worldwide to transform their teaching methods, and the experience of this transformation has been analyzed by several researchers [1–14]. Chinese medical schools introduced 12 tips that offered specific strategies to optimize teaching medical students online [1]. One of the 12 tips emphasizes the need to “promote social learning through use of online small-group learning techniques and tools.” We also conducted online PBL, including small-group discussions and presentations, which probably helped promote social learning. Reports on the use of online classes from other countries have been very informative, and we hope to apply the reported learnings to improve medical education even after the pandemic.

Although online programs were generally well accepted by medical students, reports have shown varying levels of student satisfaction with online education [2, 9, 10, 12]. Kim et al. [2] reported that 62.2% students preferred the online course over the offline course and 84.3% students wanted to continue taking online courses after the COVID-19 pandemic. However, Motte-Signoret et al. [12] reported that < 50% students felt that they received training of equivalent level and quality to that provided in their regular courses and only one-third of the included students preferred the continuation of online courses after the pandemic.

The lack of opportunities for students to interact with their peers and teachers was considered the disadvantages of online classes [1, 3, 14]. Considering that learning occurs through knowledge sharing and student interaction among their peers, attention should be paid to promote online interaction and timely feedback from teachers. In our course evaluation questionnaires, some students mentioned that they found it difficult to ask questions during online classes. Therefore, we encouraged students to use the discussion forum of Moodle, where many questions were posted by the students that allowed effective discussions between faculty and students.

Although this study evaluated the effectiveness of online education in imparting theoretical medical knowledge in junior students, medical universities must implement educational systems and methods for evaluating students’ skills and attitudes to facilitate their medical training. The acquisition of medical skills and development of attitudes are cultivated by practicing them in actual clinical clerkships. A survey from the United States [9] showed that final-year medical students did not find tele-education and e-learning as effective as face-to-face in-hospital clerkships. In our university, clinical clerkship is conducted for the 4th–6th-year students. Although clinical clerkships were cancelled for the first 4 weeks of the 2020 academic year in our university, the faculty ensured that our students practiced clerkships as much as possible under strict infection control regulations. As a result, we believe that the students could acquire practical skills that are not inferior to those who underwent clerkships before the COVID-19 pandemic.

Our study has some limitations. First, as this study included only third-year medical students who took a cardiovascular course, the effects of online education in other courses and other grades remain unknown. Second, since almost same faculty members prepared the examination question paper each year, the difficulty level of the examination for each year was considered equivalent; however, we cannot overlook the possibility of year-to-year variations in examination difficulty. The use of a standardized nationwide examination such as a computer-based test would have provided a more accurate comparison. Finally, as the online accessibility of lectures increased, student classroom attendance may have impacted their outcomes. However, considering that this course is mandatory, students were required to attend at least two-thirds of the classes to be eligible to take the examination. Therefore, student attendance was comparable, which was more than 80% in both online and face-to-face classes.

In conclusion, online classes in medical education during the COVID-19 pandemic were as successful as face-to-face classes owing to the use of the LMS and other innovations, such as online quizzes. The pandemic necessitated online education and provided an opportunity to accelerate the digital transformation of medical education. When combined with face-to-face learning, online education may positively impact the education system even after the COVID-19 pandemic.

## Data Availability

The datasets used and/or analyzed during the current study are available from the corresponding author on reasonable request.
